# Non-typhoidal, Non-paratyphoidal Salmonella Species Causing Sacroiliitis and Pyomyositis in a Healthy 19-Year-Old Athlete

**DOI:** 10.7759/cureus.18753

**Published:** 2021-10-13

**Authors:** Bhavya Narala, Maham Suhail, Vishnuveni Leelaruban, Laura Ndzelen, Yolande Mbome, Jessie Saverimuttu

**Affiliations:** 1 Internal Medicine, Richmond University Medical Center, Staten Island, USA; 2 Infectious Disease, Richmond University Medical Center, Staten Island, USA

**Keywords:** sacroiliac joint, osteomyelitis, sacroiliitis, gram negative bacteremia, infectious pyomyositis, salmonella infection

## Abstract

Salmonella is a gram-negative bacterium, subdivided into typhoidal and non-typhoidal Salmonella. It is usually caused by eating raw or undercooked meat, poultry, eggs, or egg products. The clinical manifestations of Salmonella infection can be divided into five syndromes: enterocolitis (food poisoning), enteric (typhoid) fever, bacteremia/septicemia, focal infection, and a chronic carrier state, which is usually asymptomatic. The most common clinical presentation is diarrhea. Salmonella osteomyelitis occurs most frequently in patients with sickle-cell disease; other risk factors include other hemoglobinopathies, immunocompromised status, and chronic Salmonella carrier state. The incidence of Salmonella osteomyelitis/septic arthritis in otherwise healthy individuals is rare. The duration of symptoms can range from a few months to several years, and multifocal involvement occurs in 15% of reported cases of Salmonella osteomyelitis. The symptoms of Salmonella osteomyelitis are pain and variable swelling of the affected limb; high temperatures are rarely noted. Our patient is a 19-year-old boy with no known past medical history who presented with severe right-sided sacroiliitis with extensive surrounding osteomyelitis on both sides of the sacroiliac joint with non-typhoidal, non-paratyphoidal Salmonella bacteremia.

## Introduction

Pyogenic sacroiliitis is a rare clinical entity. Bacteremia caused by non-typhoidal Salmonella is also rare but possibly fatal. Risk factors include children or younger adolescents, immunocompromised individuals (i.e. sickle cell, inflammatory bowel disease), or international travel [1]. The most common organisms that cause sacroiliitis are Staphylococcus aureus, Streptococcus species, and Pseudomonas aeruginosa [2]. Bilateral sacroiliitis caused by non-typhoidal Salmonella with bacteremia is extremely uncommon.

## Case presentation

A previously healthy, Oriental, 19-year-old male athlete with no significant past medical or family history presented to the emergency room with four days of constant excruciating pain in his right buttock; he was unable to stand or move. The onset of pain was insidious and was located in the right gluteal region, radiating medially and distally into the right hip joint. It was worsened by movement and extension of the right leg. His history revealed he had been to the emergency room one day prior for the same pain. He also complained of subjective fevers (up to 101 degrees Fahrenheit) associated with the pain. Upon arrival to the emergency room, the patient was afebrile with no leukocytosis (although a left shift was present). Due to the presenting history of pain with fever and left shift, the emergency physician decided to collect blood culture samples and sent the patient home with pain medication and muscle relaxants. He was later called back to the emergency room when blood cultures came back positive for gram-negative bacilli less than 24 hours after collection in both aerobic and anaerobic bottles. The patient denied any history of trauma, intravenous drug use, or history of immuno-suppression. He recalled that he had developed watery diarrhea two/three months prior, which lasted two to three days. He stated he was returning from a trip to Florida when he had a three-day history of loose, non-bloody bowel movements, about eight episodes per day associated with reported intermittent fevers and chills. He also remembered he had eaten rock shrimp one day prior to the onset of watery stool.

On physical examination, the patient was unable to stand from a sitting position. There was tenderness on palpation of the right gluteal region but no swelling, redness, or skin changes. Hip flexion, internal rotation, and extension could not be assessed due to severe pain. There was no paraspinal muscle tenderness but had intact sensation in both lower extremities. Vital signs during this second visit were as follows: blood pressure 111/61, pulse 96, and temperature 101.3 degrees Fahrenheit. Complete blood count revealed a white blood cell (WBC) count of 5.300/mm^3^ (77.5% polymorphonuclear leukocytes (PMN)). Laboratory studies also yielded the following results: C-reactive protein (CRP): 15.8 mg/dl (0-0.29 mg/dl) and erythrocyte sedimentation rate (ESR): 95 mm/h (0-15 mm/h). Magnetic resonance imaging (MRI) of the lumbar and sacral spine with and without contrast that was done on admission showed mild subchondral cystic change/edema along the right greater than the left sacroiliac (SI) joint, which was suspicious for sacroiliitis. There was no enhancement or abscess noted on this MRI. No other workup was conducted (such as hemoglobin electrophoresis), as there was low suspicion for sickle cell in an individual of oriental origin with no family history or anemia.

**Figure 1:**
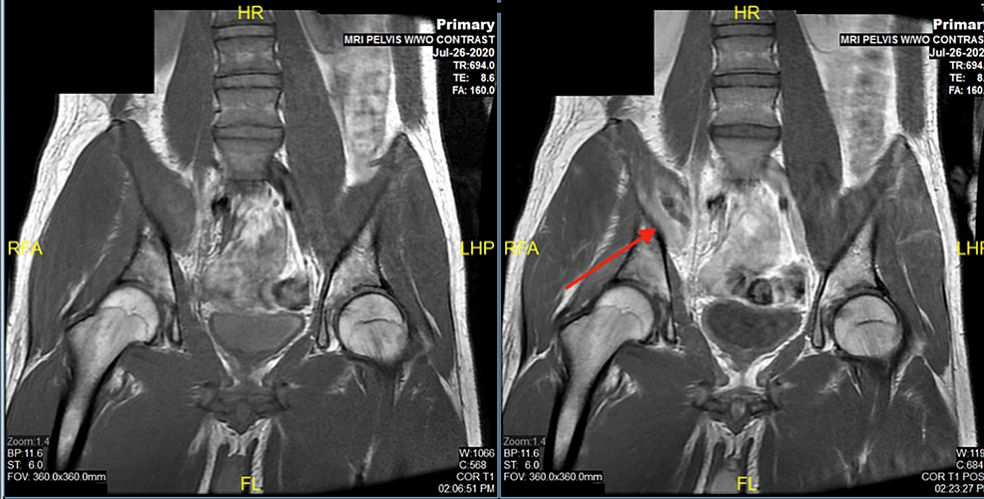
MRI of the pelvis: septic right sacroiliac joint with decompression of pus from the joint resulting in secondary pyomyositis

He was hospitalized and started on ceftriaxone 2 grams intravenously daily. Blood cultures collected during the first emergency room visit later grew Salmonella species (according to the Department of Health, Salmonella has been unable to be speciated and is untyped). MRI of the pelvis (Figure 1) was done two days later, which showed progression of findings most consistent with septic right sacroiliac joint with spontaneous decompression of pus from the joint, resulting in secondary pyomyositis. Interventional radiology was initially consulted for these findings for possible drainage. However, the team concluded that there was no safe percutaneous access to drain the right SI joint fluid due to the small size and location. In addition, attempting drainage of pyriformis muscle with minimal fluid would risk injury to the sciatic nerve and the right internal iliac artery and branches. Thus, since there was no safe window, any intervention was deferred, and the patient continued with medical treatment. His hospital course was marked by gradual resolution of the pain and regression of fever over a nine-day period. Subsequent blood cultures during admission had no growth. He was discharged home to complete a six-week course of ceftriaxone 2g IV daily. He was seen as an outpatient for follow-up four weeks after hospital discharge and reported complete resolution of symptoms. His erythrocyte sedimentation rate (ESR) improved to 13 and CRP normalized to 1.0. He had a repeat MRI, which showed similar findings. An orthopedic surgeon was consulted at this time and stated that since the patient is asymptomatic, lab values are within normal limits, and there are no subsequent sequelae, IV antibiotics were to be continued with no further intervention.

## Discussion

Pyogenic sacroiliitis caused by non-typhoidal, non-paratyphoidal Salmonella species in a healthy young adult is a rare clinical entity. Salmonella species consist of gram-negative bacilli (rods) enterobacteria [1]. Salmonella can be divided into typhoidal and nontyphoidal species. Typhoid is commonly associated with salmonella ever since typhoid fever caused many deaths occurred decades ago [3]. Most nontyphoidal Salmonella infections are caused by Salmonella (S.) Enterica subspecies, Enterica serotype enteritidis, Salmonella typhimurium, S. Newport, S. Heidelberg, and S. Javiana [2]. Human disease occurs by direct and indirect contact with numerous species of infected animals, the foodstuffs derived from them, and their excreta, contaminated meat, poultry, raw milk, eggs, egg products, and water [4]. Other reported sources include infected pet turtles and reptiles, carmine red dye, and contaminated marijuana. Salmonella infections manifest as gastroenteritis, enteric fever, bacteremia, or focal disease. The pathogenesis is not fully understood but theories suggest that activation of toll-like receptor 5 triggers an inflammatory pathway in the gastrointestinal tract leading to symptoms such as diarrhea [5].

Septic arthritis is an inflammatory condition of the joint due to invasion by an infective organism [6]. These pathogens can gain access to joint space via many routes, including hematogenous spread, through lymphatics, contiguous osteomyelitis, or direct inoculation from trauma. Common organisms causing septic arthritis in the sacroiliac joints are Staphylococcus aureus, Streptococcus species, and Pseudomonas aeruginosa [7]. Salmonella infections affecting joints and bones is rather uncommon, especially in adults. It usually occurs in patients with sickle-cell hemoglobinopathies, systemic lupus erythematosus, or those receiving immunosuppressive therapy [8]. Although any skeletal site can become infected, Salmonella infections of bone typically involve the long bones, chondrosternal junctions, knee, shoulder, hip, sacroiliac joints, and spine [9]. The disease usually affects immunocompetent adolescents, and the mean age of these patients in the literature is 18.8 years [9]. Salmonella osteomyelitis constitutes about 0.8% of all Salmonella infections found in hospitals [10]. Despite this decreased prevalence, Salmonella typhi is a common cause of osteomyelitis, especially with sickle cell anemia [11].

Sacroiliitis typically requires imaging for diagnosis. Our patient had sacroiliitis in bilateral joints. Imaging showed right greater than left, with pyomyositis of the pyriformis muscle on the right side. Most case reports showed only unilateral sacroiliitis and involved mostly the left side [12]. Plain radiographs of the sacroiliac joints are usually normal in the first two weeks of sacroiliitis [13]. Thus, MRI has become the radiologic test of choice for septic sacroiliitis and can decrease the time to diagnosis [13]. Antibiotic therapy is also significant to note. Delay in diagnosis can hinder antibiotic choice. The most commonly used antimicrobials for Salmonella osteomyelitis are chloramphenicol, third-generation cephalosporins, and fluoroquinolones [7]. Initially, our patient was empirically started on piperacillin/tazobactam but was subsequently switched to ceftriaxone. Previous reports have advised a wide range of antibiotic regimens, which include a minimum of four weeks of intravenous antibiotic therapy followed, in some cases, by a prolonged course of oral antibiotics. Nevertheless, therapy with antibiotics for four to six weeks or longer is associated with a successful outcome [14].

While this patient only required IV antibiotics, sometimes, surgical options need to be considered. According to one study, surgery generally was indicated if conservative measures failed, abscess formation was initially seen, bone destruction, septicemia, or neurological deficits, none of which our patient had [14]. Generally, the two options for surgeries involve debridement and drainage or arthrodesis of the joint. While there is some debate regarding when to choose the former versus the latter, the general consensus tends to be debridement and drainage for acute soft tissue infections or mild bone destruction. Joint arthrodesis is recommended for ill patients with worsening joint destruction and neurological deficits or problems with mobility.

Our patient originally developed severe low back pain and right buttock pain for a few days with no history of trauma. The patient did endorse having episodes of diarrhea about two to three months prior, which resolved in two to three days. While diarrhea seems like the most plausible explanation for the source of Salmonella, no other source is obvious. This, along with other factors, such as an unidentifiable species of non-typhoidal Salmonella causing bilateral sacroiliitis in an individual with no sickle cell or hemoglobinopathies, is what makes this case report so intriguing and rare.

## Conclusions

Non-typhoidal Salmonella sacroiliitis is a rare form of septic arthritis in a young healthy individual. Only a few cases have been reported worldwide. The diagnosis of sacroiliitis caused by Salmonella species requires high clinical suspicion and the key to this is a thorough anamnesis, the patient’s recollection of their medical history. In treating this disease, blood cultures should always be performed preferably before commencing antibiotic treatment. Bone scan and MRI are very sensitive for making an early diagnosis. Therapy with antibiotics for four to six weeks or longer is associated with a successful outcome. Our patient had negative blood cultures shortly after starting treatment with the appropriate antibiotic, although the clinical improvement took weeks, and repeat radiological findings lagged further. Eventually, the patient was back to baseline. To conclude, clinicians should keep the possibility of Salmonella as an etiological agent in patients with osteomyelitis/sacroiliitis, especially in those giving a history of preceding fever with or without gastrointestinal manifestations even in the absence of underlying comorbidities like hemoglobinopathies.
